# Analysis of Molecular Pretreated Tumor Profiles as Predictive Biomarkers of Therapeutic Response and Survival Outcomes after Neoadjuvant Therapy for Rectal Cancer in Moroccan Population

**DOI:** 10.1155/2020/8459303

**Published:** 2020-01-10

**Authors:** Ihsane El Otmani, Fatima El Agy, Sanae El Baradai, Laila Bouguenouch, Nada Lahmidani, Mohammed El Abkari, Dafr Allah Benajah, Imane Toughrai, Hicham El Bouhaddouti, Ouadii Mouaqit, Karim Ibn Majdoub Hassani, Khalid Mazaz, El Bachir Benjelloun, Abdelmalek Ousadden, Karima El Rhazi, Touria Bouhafa, Zineb Benbrahim, Karim Ouldim, Sidi Adil Ibrahimi, Khalid Ait Taleb, Laila Chbani

**Affiliations:** ^1^Laboratory of Biomedical and Translational Research, University of Medicine and Pharmacy of Fez, Morocco; ^2^Laboratory of Anatomic Pathology and Molecular Pathology, University Hospital Hassan II, 30070 Fes, Morocco; ^3^Unit of Medical Genetics and Oncogenetics, University Hospital Hassan II, 30070 Fes, Morocco; ^4^Department of Gastroenterology, University Hospital Hassan II, 30070 Fes, Morocco; ^5^Department of General surgery, University Hospital Hassan II, 30070 Fes, Morocco; ^6^Department of Epidemiology, University of Medicine and Pharmacy, 30070 Fes, Morocco; ^7^Department of Radiotherapy, University Hospital Hassan II, 30070 Fes, Morocco; ^8^Department of Oncology, University Hospital Hassan II, 30070 Fes, Morocco

## Abstract

Pathologic features depending on tumor response to preoperative chemoradiotherapy are important to determine the outcomes in patients with rectal cancer. Evaluating the potential predictive roles of biomarker expression and their prognostic impact is a promising challenge. We reported here the immunohistochemical staining of a panel marker of mismatch repair protein (MMR), Ki67, HER-2, and p53. Additionally, identification of somatic mutations of KRAS, NRAS, and BRAF genes were performed by direct sequencing and pyrosequencing in pretreated biopsy tissues from 57 patients diagnosed for rectal cancer. Clinical features and pathological criteria for postneoadjuvant treatment surgical resection specimen's data were collected. Immunohistochemical expression and mutational status were correlated with therapeutic response, overall survival, and disease progression. The mean age of patients was 56 years. Seven (12.3%) out of 57 patients had a complete therapeutic response. Our analysis showed that when using complete therapeutic response (Dworak 4) and incomplete therapeutic response (Dworak 3, 2, and 1) as grouping factor, high p53 expression at the pretreatment biopsy was significantly associated to an incomplete response (*p* = 0.002). For 20 and 2 out of 57, KRAS and NRAS mutations were detected, respectively. The majority of these mutations affected codon 12. KRAS mutations detected at codon 146 (A146T, A146V) was associated with the appearance of recurrence and distant metastasis (*p* = 0.019). A high expression of HER-2 corresponding to score 3+ was observed in 3 pretreatment biopsy specimens. This class was significantly associated with a short relapse-free survival (*p* = 0.002). Furthermore, the high expression of Ki67 was moderately correlated with an older age (*p* = 0.016, *r* = 0.319). In addition, this shows that high p53 expression in the pretreatment biopsy was associated with an incomplete response in surgical resection specimens after neoadjuvant treatment, and a HER-2 score 3+ can be a predictive factor of distant metastasis and local recurrence. Larger, prospective, and more studies are needed.

## 1. Introduction

Rectal cancer is one of the most malignant tumors in terms of incidence and prevalence [1, 2]. Preoperative chemoradiotherapy (CRT) combined with a total mesorectal excision (TME) is the standard treatment option for locally advanced rectal cancer (LARC) reducing rates of local recurrence [3, 4], while this approach seems to be more aggressive with patient burden, clinical toxicity resulting, as well as the financial cost care [5–7]. Indeed, the implementation of a predictive biomarker in clinical practice routine represent an urgent and strong need to identify accurately patient's therapeutic response and prognostic. However, the challenge of current medical research and numerous studies is to understand the different mechanisms of molecular pathways in malignant cells [8], as well as to comprehend the mechanisms of radiosensitivity and chemosensitivity in order to identify molecular biomarkers essential to guide therapeutic decisions and to individualize treatments of patients with rectal cancer [7, 9–13]. To our knowledge, this is the first study on the possible predictive and prognostic roles of numerous markers in rectal pretreatment samples in Moroccan population.

This study examined the expression of a panel of protein of mismatch repair protein (MMR), p53, HER-2, and Ki67 by immunohistochemistry as well as the mutational status of KRAS, NRAS, and BRAF genes by sequencing analysis and pyrosequencing. The potential predictive roles of biomarker expression and prognostic were also evaluated.

## 2. Materials and Methods

### 2.1. Patients and Pathological Examination

Material for this study was obtained from 57 pretreatment rectoscopy biopsies from patients diagnosed with rectal cancer at Hassan II University Hospital Center of Fez, between January 2012 and October 2018. Inclusion criteria were patients with biopsies of tumor fragment sufficient to perform immunohistochemical and molecular biology tests and the availability of clinical data of patients. All patients received curative therapy, including radiotherapy (45 Gy in 5 weeks) associated with concomitant chemotherapy (5-fluorouracil in continuous infusion), or exclusive radiotherapy (39 Gy/3 fractions), followed by anterior resection or abdominoperineal excision.

**Figure 1:**
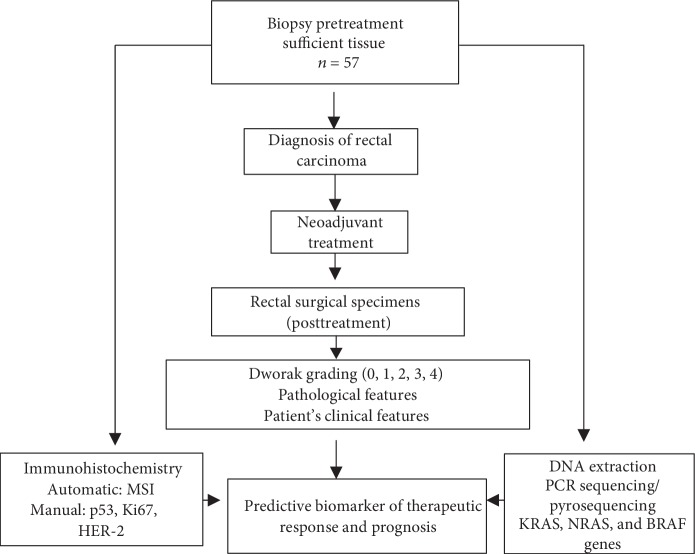
Schematic representation of evaluating predictive biomarkers of therapeutic response and prognosis.

Formalin-fixed paraffin-embedded (FFPE) biopsy tissue blocks were fixed in 10% formalin and selected for immunohistochemistry (IHC) and DNA isolation, see Figure 1.

Histological slides based on a hematoxylin and eosin-stained slide were evaluated by a gastrointestinal pathologist. Histological parameters were investigated and performed according to the staging criteria of the American Joint Committee on Cancer,7th edition (AJCC) [14] (histological type, tumor differentiation, tumor regression grade with the Dworak grading, postneoadjuvant treatment TNM stage (ypTNM), lymph node status, and other clinicopathological characteristics). Therapeutic response on surgical resected specimens was defined according to two methods. The first method, in which we obtained two groups, was performed according to Dworak tumor regression; a group of complete response corresponding to Dworak 4, and a group of incomplete response corresponding to Dworak 3, 2, and 1. The second method depends on the percentage of tumor regression and defines a group of good responders with a percentage higher than 50% versus the group of nonresponders with a percentage of tumor regression of below 50%.

Data registered included demographic details, neoadjuvant treatment details, type and results of surgery, pathology reports, and cancer outcome.

### 2.2. Immunohistochemistry

Multiple fine sections (4 ym) were cut and mounted on superfrost glass slides from the biopsy tissue blocks for subsequent immunostaining. Automatic immunohistochemistry (Roche Ventana BenchMark ULTRA Slide Stainer) was used for determining the MSI status, and the slides were stained with mouse monoclonal antibodies specific for each MMR protein: hMLH1 (clone-M1), hMSH2 (clone G219-1129), hMSH6 (clone-44), and hPMS2 (clone A16-4). Manual immunohistochemistry was performed according to the manufacturer's protocol for other markers. Slides incubated at 56°C during the night preceding the technique were deparaffinized using toluene, rehydrated with serial alcohol, and distilled water. EnVision™ FLEX, High pH (Link), DAKO system, was used in all following steps. Unmasking was performed by immersion slides in target retrieval solution 0.001 M (pH 9.0) at a preheated temperature of 65°C and then a target final temperature set at 95°C during 20 min. Slides were immersed in a wash buffer for 5 min and treated with peroxidase-blocking reagent for 3 min to block endogenous peroxidase activity. Specific antibodies for each protein were used. For staining nuclear Ki67 protein, a DAKO mouse monoclonal antibodies (clone MIB-1) was used at the dilution of 1/2, and the tissue section was then incubated during 1 hour at room temperature. Rabbit polyclonal antibodies to c-erbB-2 was used to evaluate HER-2 expression at the dilution of 1/1000 and incubated during 40 min at room temperature. Mouse monoclonal antibodies (clone DO-7) were used to stain p53 protein during 40 min. HRP system detection was used during 30 min followed by 5 min incubation with 3,3′-diaminobenzidine for color reaction. A counterstaining using hematoxylin for 2 min was performed. Final serial ethanol washes were used followed by a mounting of the slats with a specific glue.

The slides were analyzed, and the staining of each marker was evaluated according to a specific manner by an experienced gastrointestinal pathologist.

MSI status was assessed by analyzing the presence or absence of MMR protein expression in tumor section. Normal colonic epithelium adjacent to the tumor area was used as a positive control.

Ki67 staining was evaluated, and the cell proliferation index was calculated as a percentage of stained cells relative to all cells examined irrespective of intensity. Two groups of patients were determined: the first group expressed a proliferation index of less than 50%, and the second group expressed a proliferation index more than 50%. The analysis of HER-2 membrane immunostaining was based on a semiquantitative histologic score. Score 0 corresponding to an absence of staining, score (1+) corresponding to a weak staining, score (2+) corresponding to a moderate staining, and score (3+) corresponding to a strong staining [15]. The assessment of p53 immunostaining classes was depending on the percentage of stained cells relative to all cells examined in tumor section: class 0, 0-20%, class 1, 20-49%, and class 2, 50-100%.

### 2.3. Tumor DNA Preparation and Analysis

FFPE biopsy blocks were cut into 3 to 4 fine slices (8-10 ym) and transferred to an Eppendorf tube, to ensure tumor cell enrichment. Tumor areas were surrounded and tumor cell percentage determined on a haematoxylin and eosin-stained slide using a microscopic observation. DNA isolation was performed subsequently according to the manufacturer's protocol provided with the Invitrogen DNA/RNA FFPE kit. Briefly, microdissected sample was deparaffinated with toluene. Ethanol was used to remove the existing toluene in the sample after centrifugation. Digestion buffer and proteinase K were added to the sample and then incubated overnight at 56°C, followed by serial washes and a final elution. The total DNA was measured using the Qubit 3.0 fluorometer.

KRAS exon 2, 3, and 4 mutations, NRAS exon 2, 3, and 4 mutations, and exon 15 of BRAF mutation were analyzed using a direct sequencing performed after a carried PCR with specific primers (see [Supplementary-material supplementary-material-1]) in 20 *μ*L reaction containing 2 *μ*L DNA of a concentration of 11 ng/*μ*L. Pyrosequencing was performed using the PyroMark therascreen KRAS for sample with cell tumor percentage of less than 30%.

### 2.4. Statistical Analysis

Clinicopathological features were assessed using the *χ*^2^ test and Fisher's test as appropriate. Survival curves were performed using the Kaplan-Meier method. Statistical analysis was carried out using the statistical package SPSS V.21. A *p* value of <0.05 was considered statistically significant.

### 2.5. Follow-Up

The overall survival (OS) duration was defined as the time between the date of diagnosis and the date of death by any causes or last follow-up visit. The relapse-free survival (RFS) duration was defined as the time between the date of diagnosis and the date of first distant or local disease recurrence or last follow-up. Follow-up examinations were routinely taken after neoadjuvant and surgical treatment. A colonoscopy was performed after the first surgery and then once at the interval of 2 or 3 years. The MRI or contrast-enhanced CT of the abdomen and pelvis were performed every 6 months. Additionally, blood examination, physical examination, and necessary routine tests were carried out.

### 2.6. Ethics

The study protocol was approved by the local ethics committee at the Faculty of Medicine and Pharmacy of Fez and Hassan II University Hospital under the number 26/17 and was conducted in accordance with the ethical standards of the Declaration of Helsinki. Informed consents were obtained from all patients.

## 3. Results

### 3.1. Patient and Molecular Characteristics

Fifty-seven consecutive patients who were diagnosed with rectal cancer were identified and included in the database. The characteristics of the patients are presented in Table 1. 25 (43.9%) were female and 32 were (56.1%) male with a sex ratio of 0.78. The mean age was 56 years (25-82). At the time of diagnosis, the majority of the tumors were located in the low and middle rectum (low: 45.6, middle: 45.6%, high: 5%). Chemoradiotherapy was the most common neoadjuvant treatment with a rate of 68.4%, followed by exclusive radiotherapy (31.6%). 47 patients underwent anterior resection (82.5%), while abdominoperineal resection was performed in only 10 patients (17.5%).

54 (94.7%) preoperative biopsy samples were diagnosed for adenocarcinoma histologic type and 3 (5.3%) of them were diagnosed for mucinous and signet ring carcinoma. 29 (50.9%) tumors were well differentiated, 25 (43.9%) tumors were moderately differentiated, and 3 (5.3%) were poor and undifferentiated. Out of 57 patients, 7 (12.3%) achieved pathologic complete response. Analyzing therapeutic response according to the percentage of tumor regression showed 36 (63.2%) patients with more than 50% response versus 21 (36.8%) with less than 50% of therapeutic response. A perineural invasion was present in 7 (12.3%). Vascular invasion was identified in 5 (8.8%) tumors. Pathological T stage was classified as ypT0 in 7 patients, ypT1 in 3 patients, ypT2 in 21 patients, ypT3 in 25 patients, and ypT4 in 1 patients. 38 tumors were staged ypN0, and 19 tumors were staged as positive nodal status.

### 3.2. RAS and BRAF Mutational Status and Pathological Response

RAS mutation status is shown in Table 2. The KRAS mutations were found in 20 (38.59%) of 57 patients. In addition, it was detected at codon 12 (G12V. G12D, and G12C) in 14 patients, at the codon 13 (G13D) in 2 patients, and at the codon 146 at 4 patients. NRAS mutations represent 9.09% of all mutations and were detected at codon 12 (G12D) of exon 2 in 1 patient and codon 61 (Q61L) of exon 3 in a second patient. No mutation was detected in BRAF exon 15 of all patients.

### 3.3. Association between Marker Expression Level and Complete Pathologic Response

**Figure 2:**
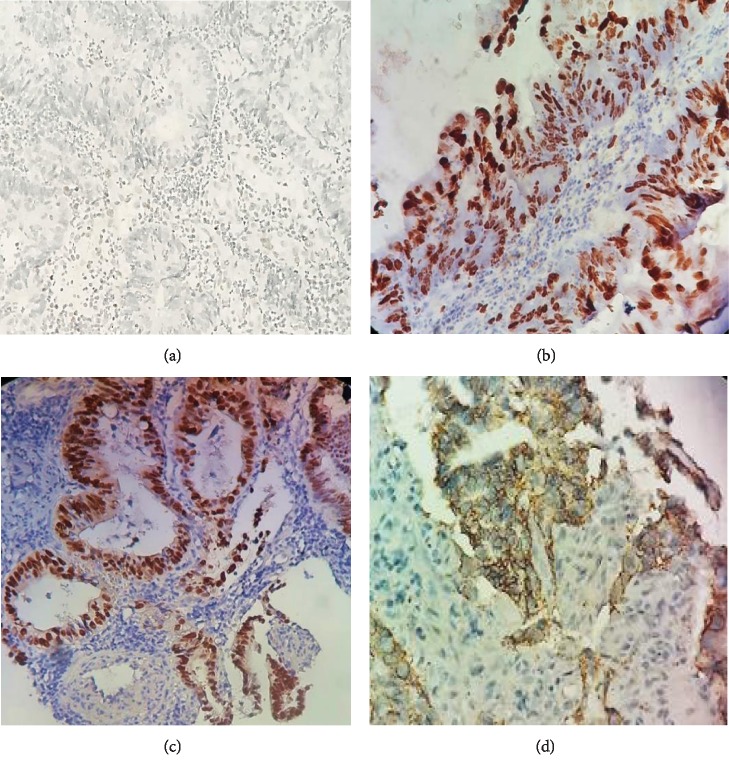
Immunohistochemical expression of pretreatment biomarkers. (a) MSH6 expression showing an unstableness (×400). (b) Expression of 80% of nuclear Ki67 (×400). (c) Nuclear expression of p53 (×20). (d) Expression of HER-2 showing a score 3+ (×20).

Table 3 summarizes the association between molecular biomarkers and therapeutic response as grouping by complete and incomplete response.  Figure 2 shows examples of results obtained using immunohistochemical staining of the different biomarkers of MSH-6, Ki-67, P53, and HER-2.

Tables [Supplementary-material supplementary-material-1] and [Supplementary-material supplementary-material-1] in Supplementary Materials recap the association between molecular biomarkers and therapeutic response as grouping by four Dworak grades (1, 2, 3, and 4) and the percentage of therapeutic effect, respectively. There was no significant correlation between Ki-67, MSI, and HER-2 expression in the preoperative samples and the therapeutic response. While the p53 high expression was present in 40 (93%) of samples with an incomplete response versus only 3 (7%) with complete response, the difference between the two groups was statistically significant (*p* = 0.002).

### 3.4. Marker Expression and Clinicopathological Features

**Figure 3:**
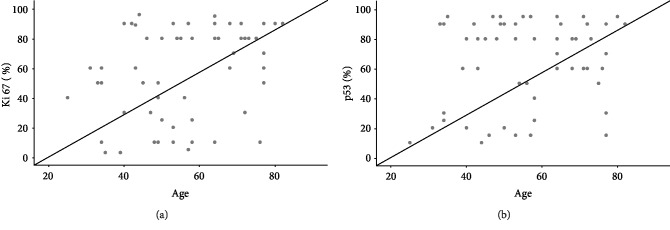
Correlation between Ki67 and p53 percentage expression and the age of patients. (a) A moderate correlation observed between age of patients and the percentage expression of Ki67 in pretreated biopsies tissues (*p* = 0.016). (b) No correlation observed between a patient's age and the percentage of p53 (*p* = 0.151).

The relation between clinicopathological characteristics and the level of expression of markers is illustrated in [Supplementary-material supplementary-material-1] in Supplementary Materials. There was no significant correlation between immunohistochemistry expression markers and the demographic and pathologic features. A moderate positive correlation was found between the percentage of Ki67 expression and the age of patients (*r* = 0.319, *p* = 0.019, see Figure 3).

Analysis of codon's mutations of KRAS and NRAS genes and their association with clinicopathological criteria (see [Supplementary-material supplementary-material-1] in Supplementary Materials) showed a significant association between KRAS mutations detected at codon 146, corresponding to the mutations of A146T and A146V in pretreatment biopsy specimens, and the presence of both recurrence and distant metastasis (*p* = 0.019). While other codons did not show a significant difference.

### 3.5. Association between Marker Expression Level and Survival

**Figure 4:**
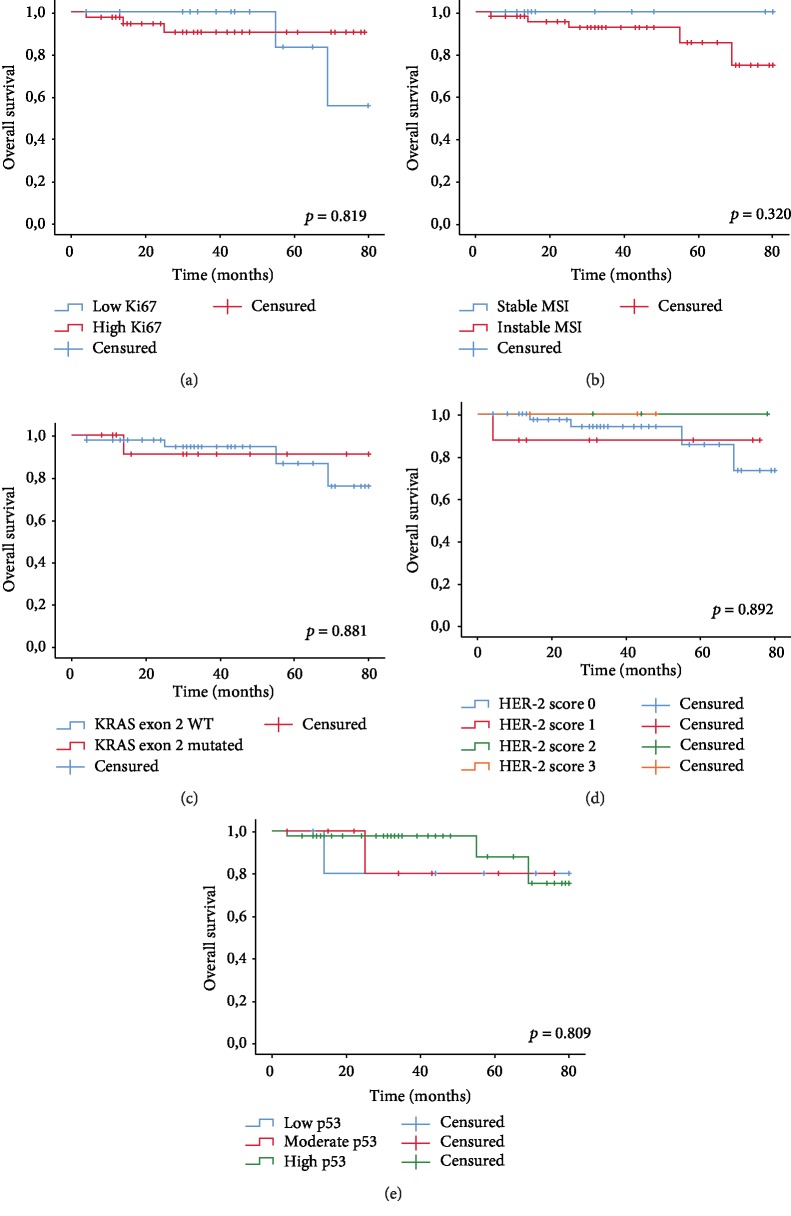
Overall survival functions. (a) Overall survival function according to Ki67. (b) Survival function according to MSI. (c) Survival function according to KRAS exon 2 mutations. (d) Survival function according to HER scoring. (e) Overall survival function according to p53 expression.

Kaplan-Meier curves did not reveal a significant association between OS and biomarker expressions of Ki67, MMR, and RAS mutations, HER-2, and P53 (*p* = 0.819, *p* = 0.320; *p* = 0.881, *p* = 0.892, *p* = 809, respectively, see Figures 4(a)-4(e)).

**Figure 5:**
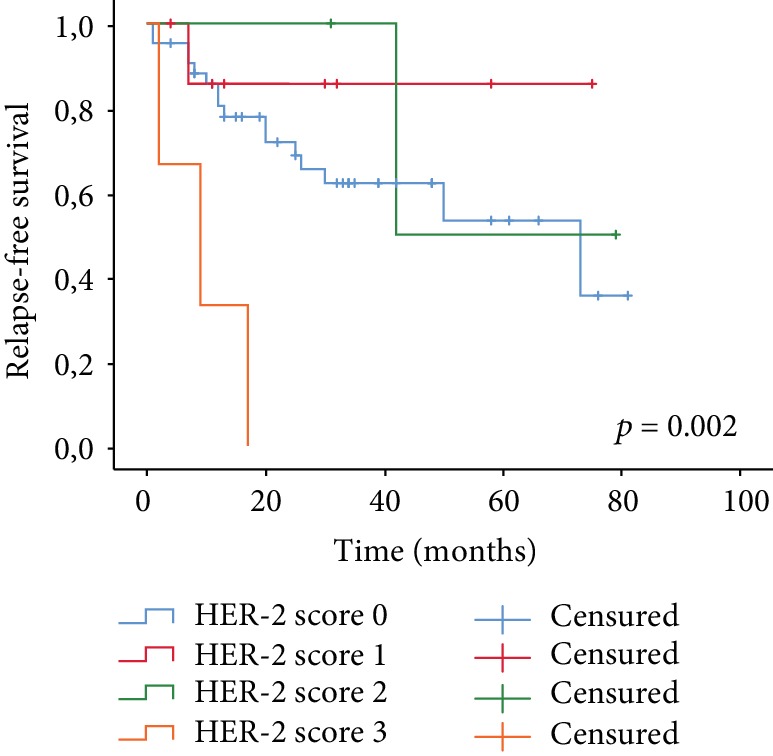
Relapse-free survival function according to HER-2 scoring.

A high expression of HER-2 corresponding to score 3+ was observed in pretreatment biopsy specimens. This class was significantly associated with a short RFS (*p* = 0.002, see Figure 5), while other markers did not show any association with RFS.

## 4. Discussion

In the present study, we investigated a panel of 7 biomarkers reportedly associated with tumor responses to preoperative CRT in rectal cancer [9, 12]. Nowadays, numerous molecules have been studied as potential biomarkers of radiosensitivity in rectal cancer. Although promising, the results have often been controversial and have not resulted in the establishment of either individual biomarkers or a panel of biomarkers that could be used to distinguish rectal tumors that are radiosensitive from those that are not [13].

p53 protein intervenes in the regulation of cell proliferation, differentiation, repair of DNA damage, and apoptosis [16]. In our study, the high expression of p53 was considered as a predictive marker of incomplete therapeutic response. Hur et al. reported the same result in about 81 tumors analyzed prior to neoadjuvant treatment [17]. While numerous trials have suggested that a positive expression of p53 can also be related to the hyperexpression of a wild-type gene and to the stabilization of the normal p53 protein by binding with the mdm2 protein or with other proteins [18, 19], the American Joint Committee on Cancer considered that the status p53 is associated with the category of markers whose prognostic value is not sufficiently established, and for which additional investigations are required [20].

Furthermore, microsatellite profile of colorectal cancer (CRC) provides useful and interesting information of patient's prognosis [21]; thus, a better overall survival rate was related to microsatellite unstable tumors [22]. MMR proteins' role is to identify mismatch in DNA sequences occurring in DNA replication process and then to repair them [23]. Mutation accumulation in a cell is due to an inactivating mutation in any of these genes coding for MMR protein; this finally leads to a malignant transformation process [24–26]. Results of a previous study including 209 consecutive patients with rectal cancer, of Huh et al., showed that MSH6 protein expression is an independent predictor for overall survival in pretreatment biopsy tissue, although it proved that MMR protein expression is not a predictive factor of radiation response [11]. In our study, MMR protein expression analyzed separately did not show any relation between outcomes of patients and their response to neoadjuvant treatment.

Ki67 protein is a cell proliferation marker, which can be detected in all stages and cycles of cell differentiation [27]. The diffuse expression of Ki67 was associated with a response to chemotherapy. In our context, no association has been observed between the different therapeutic response groups and the expression of Ki67. Bertolini et al. did not find an association of Ki67 expression and therapeutic response in their study, as reported by other several authors [28–31], with the exception of Huerta et al. who showed in their analysis conducted in 2010 that the expression of Ki67 is an independently predictive factor of the histological response after neoadjuvant treatment [32]. Although Ki67 reflects the active cell cycle, it does not necessarily quantify the speed of the cell cycle process which can be the critical factor of sensitivity to the chemotherapy [33].

Epidermal growth factor receptor 2 (HER-2) has tyrosine kinase activity and plays a role in cell growth, division, and survival as described by Meng et al. [34]. HER-2 positive expression corresponding to score3+ (excluding equivocal score 2) in our study was detected in 5.3% of all cases; this was confirmed by several other studies with a positive expression in 0.5% to 49.6% in CRC [35–37], while this expression was not significantly associated with therapeutic response as reported by the study of Kwon et al. [38]. We also found that a strong membrane expression (score 3+) of HER-2 was associated with a short relapse time of patients. Other studies have shown a similar survival outcome, HER-2 hyperexpression has been associated with low overall survival and disease-free survival in patients with colorectal cancer reported by Heppner et al. and Lim et al. [39, 40]. Kwon et al. recommended that the use of a HER-2 gene amplification is important even in cases with an HER-2 IHC score of low expression (scores 0 and 1), as 7.7% of HER-2 OHC and 11.1% of IHC HER-2 1 showed an amplification of the HER-2 gene [38].

Kirsten-ras (Ki-ras) oncogene mutations are involved in the mechanism transformation of adenoma to carcinoma in colorectal cancer. KRAS mutation is responsible for the essential activation of the KRAS protein which results in dysregulation of downstream processes and difficulties on cell growth control [41, 42]. An acceleration in tumor growth and an earlier appearance of distant metastasis as well as resistance to antiepidermal growth factor receptor are related to KRAS mutations [42–45]. 85% of KRAS mutations are detected in codon 34 and codon 35 [42, 43]. Several trials have shown that patient's prognosis depends on the type of codon mutations, such as codon 12 mutations; the KRAS G12V mutations are more aggressive than KRAS G12D mutations in term of prognosis [43].

In the present study, among 57 biopsies analyzed for mutations in NRAS and KRAS genes, the majority of KRAS mutations were found in codons 34 and 35 as mentioned in numerous studies [42, 46–48]. A similar study was performed on 47 patients and showed that 12 of 14 patients had mutations in codon 35 [45]. Lee et al. reported that the KRAS mutation status in locally advanced rectal cancer is not a predictive factor of tumor response and survival of patients after preoperative chemoradiotherapy [49]. The present study is interesting because it highlights the predictive role of 146 codon mutations (A146T, A146V) in pretreated biopsies on the appearance of relapse in patients with rectal cancer treated with neoadjuvant therapy. However, RAS mutation was not a significant predictor for tumor response to neoadjuvant therapy and survival outcomes. In our study, V600E BRAF mutations were absent in pretreated tumor biopsies; this confirmed the data from other studies [50].

The present study has some limitations. The limited number of patients and the retrospective nature of this study represent major limitations for the clinical applicability of this information. Further investigations are required to clarify the influence of these biomarkers on rectal cancer features after neoadjuvant chemoradiotherapy.

## 5. Conclusion

In summary, our study showed that a high p53 expression at the pretreatment biopsy tumors was a predictive biomarker to an incomplete response to neoadjuvant chemoradiotherapy. In addition, the presence of mutations in codon 146 of the *KRAS* gene (A146T, A146V) was found to be a predictor of relapse-free survival. Furthermore, a high HER-2 expression of score 3+ can be a predictive factor of distant metastasis and local recurrence. Larger, prospective, and more studies are needed.

## Figures and Tables

**Table 1:** Clinicopathological and molecular characteristics of the study.

	Patients (*N* = 57 (%))
Demographic characteristics	
Median age, years	56 (28-81)
Gender	
Female	25 (43.9)
Male	32 (56.1)
Age	
*<*50	23 (40.4)
≥50	34 (59.6)
Clinicopathological characteristics	
Location of tumor	
High	5 (8.8)
Middle	26 (45.6)
Low	26 (45.6)
Type of neoadjuvant treatment	
Chemoradiotherapy	39 (68.4)
Radiotherapy	18 (31.6)
Surgical procedure	
Abdominoperineal excision	10 (17.5)
Anterior resection	47 (82.5)
R0/R1 status	
R0	52 (91.2)
R1	5 (8.8)
Histologic type	
Adenocarcinoma	54 (94.7)
Mucinous carcinoma/signet ring carcinoma	3 (5.3)
Degrees of differentiation	
Well	29 (50.9)
Moderate	25 (43.9)
Poor/undifferentiated	3 (5.3)
Therapeutic response according to the percentage of tumor regression	
*<*50%	21 (36.8)
≥50%	36 (63.2)
Therapeutic response according to Dworak	
Complete response	7 (12.3)
Incomplete response	50 (87.7)
Dworak	
1	8 (14)
2	20 (35.1)
3	22 (38.6)
4	7 (12.3)
Perineural invasion	
Yes	7 (12.3)
No	50 (87.7)
Vascular invasion	
Yes	5 (8.8)
No	52 (91.2)
ypN	
N0	38 (66.7)
N+	19 (33.3)
ypT	
T0	7 (12.3)
T1	3 (5.3)
T2	21 (36.8)
T3	25 (43.9)
T4	1 (1.8)
Survival	
Death	5 (8.8)
Lost to follow-up/alive	52 (91.2)
Local and distant relapse	
No	36 (63.2)
Yes	21 (36.8)
Molecular characteristics	
Ki67	
*<*50	18 (31.6)
≥50	39 (68.4)
MSI	
Stable	23 (40.4)
Unstable	11 (19.3)
BRAF exon 15 (V600E)	
Wild type	47 (100)
Mutated	0 (0)
KRAS 2	
Wild type	41 (71.9)
Mutated	16 (28.1)
HER-2 scoring	
0	43 (75.4)
1	8 (14)
2	3 (5.3)
3	3 (5.3)
p53	
*<*20	6 (10.5)
20-50	8 (14)
≥50	43 (75.4)

**Table 2:** Type of RAS mutation detected in our study.

Gene	Exon	Codon change	Nucleotide change	Protein change	N
KRAS	2	12	GGT-GTT	p.G12V	4/57
	2	12	GGT-GAT	p.G12D	7/57
	2	12	GGT-TGT	p.G12C	3/57
	2	13	GGC-GGA	p.G13D	2/57
	4	146	GCA-ACA	p.A146T	2/57
	4	146	GCA-CCA	p.A146V	2/57

NRAS	2	12	GGT-GAT	p.G12D	1/47
	3	61	GAA-GTA	p.Q61L	1/57

**Table 3:** Association between therapeutic response and molecular biomarkers.

	Total	Therapeutic response		*p* value
		Incomplete response, *N* (%)	Complete response, *N* (%)	
Ki67	57			0.620
*<*50		16 (88.9)	2 (11.1)	
≥50		34 (87.2)	5 (12.8)	
MSI	57			0.220
Unstable		9 (81.8)	2 (18.2)	
Stable		41 (89.1)	5 (10.9)	
MSH6				
Stable	57	47 (87)	7 (13)	0.100
Unstable		3 (100)	0 (0)	
MSH2	57			
Stable		48 (88.9)	6 (11.1)	0.330
Unstable		2 (66.7)	1 (33.3)	
MLH1	57			
Stable		48 (88.9)	6 (11.1)	0.330
Unstable		2 (66.7)	1 (33.3)	
PMS2	57			
Stable		47 (88.7)	6 (11.3)	0.417
Unstable		3 (75)	1 (25)	
KRAS exon 2	57			
Wild type		35 (85.4)	6 (14.6)	0.660
Mutated		15 (93.8)	1 (6.3)	
KRAS exon 3	50			
Wild type		44 (88)	6 (12)	^∗^
Mutated		0 (0)	0 (0)	
KRAS exon 4	51			
Wild type		42 (89.4)	5 (10.6)	1.000
Mutated		4 (100)	0 (0)	
NRAS exon 2	47			
Wild type		41 (89.1)	5 (10.9)	1.000
Mutated		1 (100)	0 (0)	
NRAS exon 3	57			
Wild type		49 (87.5)	7 (12.5)	1.000
Mutated		1 (100)	0 (0)	
NRAS exon 4	47			^∗^
Wild type		40 (90.9)	4 (9.1)	
Mutated		0 (0)	0 (0)	
HER-2 scoring	57			
0		37 (86)	6 (14)	0.810
1		7 (87.5)	1 (12.5)	
2		3 (100)	3 (100)	
3		3 (100)	0 (0)	
p53	57			0.002
*<*20		6 (100)	0 (0.0)	
20-50		4 (50)	4 (50)	
≥50		40 (93)	3 (7)	

^∗^ *p* value was not calculated because the variable is constant.

## Data Availability

The data used to support the findings of this study are available from the corresponding author upon request.

## Supplementary Materials

Supplementary Materials Table S1: the sequence of primers used in PCR. Table S2: association between TRG Dworak regression and biomarkers. Table S3: association between the percentage of therapeutic response and biomarkers. Table S4: association between clinicopathological characteristics and immunohistochemical biomarkers. Table S5: association between clinicopathological characteristics and KRAS and NRAS mutations' relapse-free survival probability.
