# Supplementary material for: Analysis of Molecular Pretreated Tumor Profiles as Predictive Biomarkers of Therapeutic Response and Survival Outcomes after Neoadjuvant Therapy for Rectal Cancer in Moroccan Population
Source: Dis Markers. 2020 Jan 10;2020:8459303. doi: 10.1155/2020/8459303 (PMC6977322; doi:10.1155/2020/8459303)
Supplement: Supplementary Materials — Table S1: the sequence of primers used in PCR. Table S2: association between TRG Dworak regression and biomarkers. Table S3: association between the percentage of therapeutic response and biomarkers. Table S4: association between clinicopathological characteristics and immunohistochemical biomarkers. Table S5: association between clinicopathological characteristics and KRAS and NRAS mutations' relapse-free survival probability. [file 8459303.f1.pdf]

Supplementary Materialsfor:

Analysis of Molecular Pretreated Tumor Profiles as Predictive Factors of Therapeutic Response and Survival Outcomes After Neoadjuvant Therapy For Rectal Cancer in Moroccan Population

Ihsane El Otmani [1,2,3]*, Fatima El Agy [1,2], Sanae El Baradai[2], Laila Bouguenouch[3], Nada Lahmidani[4], Mohammed El Abkari[4], Dafr Allah Benajah[4], Imane Toughrai[5], Hicham El Bouhaddouti[5], Ouadii Mouaqit[5],Ibn Majdoub Hassani Karim[5], Khalid Mazaz[5], El Bachir Benjelloun[5],Abdelmalek Ousadden[5],Karima El Rhazi[6], Touria Bouhafa[7], Zineb Benbrahim[8], Karim Ouldim[3], SidiAdil Ibrahimi[5],Khalid Ait Taleb[5],Laila Chbani[1,2]

Table S1:The sequence of primers used in PCR

Sequence of primers F/R : (5'>3')
**KRAS gene**
**Exon 2**<br>*F : GGTGGAGTATTTGATAGTGTA*<br>*R: GGTCCTGCACCAGTAATATGCA*<br>**Exon 3**<br>*F : AGTAAAAGGTGCACTGTAATA*<br>*R : CTCCTTAATGTCAGCTTATTAT*<br>**Exon 4**<br>*F: CTCTGAAGATGTACCTATGGTC*<br>*R: CAGTGTTACTTACCTGTCTTGT*
**NRAS gene**
**Exon 2**<br>*F : ATTTGCCATAAATAAAGGC*<br>*R : TAAATAAATTTGCCATA*<br>**Exon 3**<br>*F: ACCCTCCACACCCCCAGGATT*<br>*R: CTGTAGAGGTTAATATCCGCA*<br>**Exon 4**<br>*F: CCCGTTTTTAGGGAGCAGATTA*<br>*R: CCAGAGTTAATCAACTGATGC*
**BRAF gene**
**Exon 15**<br>*F: TGCTTGCTCTGATAGGAAAATG*<br>*R: GTAACTCAGCAGCATCTCAGGG*

F, Forward. **R**, Reverse. **PCR**, Polymerase chain reaction

Table S2: Association between TRG Dvorak regression and biomarkers

	Dvorak				p value
	1	2	3	4	
Ki 67					
**<50**	1(5.6)	9(50)	6(33.3)	2(11.1)	0.357
**⩾50**	7(17.9)	11(28.2)	16(41.1)	5(12.8)	
MSI					
**Stable**	7(15.2)	14(30.4)	20(43.5)	5(18.2)	0.312
**unstable**	1(9.1)	6(54.5)	2(18.2)	2(10.9)	
KRAS exon 2					
**Wild type**	6(14.6)	15(36.6)	14(34.1)	6(14.6)	0.673
**Mutated**	2(12.5)	5(31.3)	8(50)	1(6.3)	
HER 2 scoring					
**0**	7(16.3)	13(30.2)	17(39.5)	6(14)	0.703
**1**	0(0)	5(62.5)	2(25)	1(12.5)	
**2**	1(33.3)	1(33.3)	1(33.3)	0(0)	
**3**	0(0)	1(33.3)	2(66.7)	0(0)	
P53					
**<20**	0(0)	3(50)	3(50)	0(0)	0.026
**20-50**	1(12.5)	2(25)	1(12.5)	4(50)	
**⩾50**	7(16.3)	15(34.9)	18(41.9)	3(7)	

Table S3: Association between the percentage of therapeutic response and biomarkers

	Therapeutic response N=57		P value
	Non responder group <50% of therapeutic response	Responder group ⩾50% of therapeutic response	
Ki 67			0.527
**<50**	7(39.9)	11(61.1)	
**⩾50**	14(35.9)	25(64.1)	
MSI			
**Stable**	16(34.8)	30(65.2)	0.372
**instable**	5(45.5)	6(54.5)	
KRAS exon 2			
**Wild type**	16(39)	25(61)	0.410
**Mutated**	5(31.3)	11(68.8)	

HER 2 scoring			
**0**	16(37.2)	27(62.8)	0.998
**1**	3(37.5)	5(62.5)	
**2**	1(33.3)	2(66.7)	
**3**	1(33.3)	2(66.7)	
P53			
**<20**	3(50)	3(50)	0.628
**20-50**	2(25)	6(75)	
⩾50	16(37.2)	27(62.8)	

TableS4: Association between clinicopathological characteristics and Immunohistochemical biomarkers

	MSI		p	Ki-67		p	HER-2				p	P-53			p
	stable	unstable		Low	high		Score 0	Score 1	Score 2	Score 3		low	moderate	high	
Gender **Female**	26(56.5)	6(54.5)	0.583	11(61.1)	21(53.8)	0.412	22((51.2)	6(75)	3(100)	1(33.3)	0.206	5(83.3)	3(37.5)	24(55.8)	0.231
**Male**	20(43.5)	5(45.5)		7(38.9)	18(46.2)		21(48.8)	2(25)	0(0)	2(66.7)		1(16.7)	5(62.5)	19(44.2)	
Age **<50**	17(37)	6(54.5)	0.232	9(50)	14(35.9)	0.236	18(41.9)	2(25)	3(100)	0(0)	**0.063**	3(50)	4(50)	16(37.2)	0..698
**⩾50**	29(63)	5(45.5)		9(50)	25(64.1)		25(58.1)	6(75)	0(0)	3(100)		2(50)	4(50)	27(62.8)	
Histologic type			0.519			0.688					0.794				0.597
**Adenocarcinoma**	43(93.5)	11(100)		17(94.4)	37(94.9)		40(93)	8(100)	3(100)	3(100)		6(100)	8(100)	40(93)	
**Mucinous carcinoma/signet ring carcinoma**	3(6.5)	0(0)		1(5.6)	2(5.1)		3(7)	0(0)	0(0)	0(0)		0(0)	0(0)	3(7)	
Degrees of differentiation			0.562			0.446					0.958				0.801
**Well**	24(52.2)	5(45.5)		9(50)	20(51.3)		21(48.8)	4(50)	2(66.7)	2(66.7)		3(50)	3(37.5)	23(53.5)	
**Moderate**	19(41.3)	6(54.5)		9(50)	16(41)		19(44.2)	4(50)	1(33.3)	1(33.3)		3(50)	4(50)	18(41.9)	
**poor/undifferenciated**	3(6.5)	0(0)		0(0)	3(7.7)		3(7)	0(0)	0(0)	0(0)		0(0)	1(12.5)	2(4.7)	
Perineural invasion			0.610			0.667					0.813				0.621
**Yes**	5(10.9)	2(18.2)		3(16.7)	4(10.3)		6(14)	1(12.5)	0(0)	0(0)		0(0)	1(12.5)	6(14)	
**No**	41(89.1)	9(81.8)		15(83.3)	35(89.7)		37(86)	7(87.5)	3(100)	0(0)		6(100)	7(87.5)	37(86)	
Vascular invasion **Yes**	3(18.2)	2(18.2)	0.244	2(11.1)	3(7.7)	0.646	5(11.6)	0(0)	0(0)	0(0)	0.618	0(0)	0(0)	5(11.6)	0.410
**No**	43(93.5)	9(81.8)		16(88.9)	36(92.3)		38(88.4)	8(100)	3(100)	3(100)		6(100)	8(100)	38(88.4)	

ypN	**N0**	31(67.4)	7(63.6)	0.537	11(61.1)	27(69.2)	0.377	29(67.4)	4(50)	3(100)	2(66.7)	0.473	4(66.7)	7(87.5)	27(62.8)	0.396
	**N+**	15(32.6)	4(36.4)		7(38.9)	12(30.8)		14(32.6)	4(50)	0(0)	1(33?3)		2(33.4)	1(12.5)	16(37.2)	
ypT	**T0**	5(10.9)	2(18.2)	0.086	2(11.1)	5(12.8)	0.515	6(14)	1(12.5)	0(0)	0(0)	0.991	0(0)	4(50)	3(7)	0.091
	**T1**	2(4.3)	1(9.1)		0(0)	3(7.7)		3(7)	0(0)	0(0)	0(0)		0(0)	0(0)	3(7)	
	**T2**	20(43.5)	1(9.1)		9(50)	12(30.8)		15(34.9)	3(37.5)	2(66.7)	1(33.3)		3(50)	2(25)	16(37.2)	
	**T3**	19(41.3)	6(54.5)		7(38.9)	18(46.2)		18(41.9)	4(50)	1(33.3)	2(66.7)		3(50)	2(25)	20(46.5)	
	**T4**	0(0)	1(0)		0(0)	1(2.6)		1(2.3)	0(0)	0(0)	0(0)		0(0)	0(0)	1(2.3)	
Recurrence	**No**	44(96.7)	1(9.1)	0.481	16(88.9)	38(97.4)	0.232	40(93)	8(100)	3(0)	3((100)	0.794	5(100)	20(95.2)	29(93.5)	0.829
	**Yes**	2(4.3)	10(90.9)		2(11.1)	1(2.6)		3(7)	0(0)	0(0)	0(0)		0(0)	1(4.8)	2(6.5)	
Metastasis	**No**	29(63)	8(72.7)	0.409	14(77.8)	23(59)	0.139	28(65.1)	7(87.5)	2(66.7)	0(0)	0.062	3(50)	6(75)	28(65.1)	0.624
	**Yes**	17(37)	3(27.3)		4(22.2)	16(41)		15(34.9)	1(12.5)	1(33.3)	3(100)		3(50)	2(25)	15(34.9)	
Metastasis or/and recurrence				0.357			0.254					0.066				0.628
**No**		28(60.9)	8(72.7)		13(72.2)	23(59)		27(62.8)	7(87.5)	2((66.7)	0(0)		3(50)	6(75)	27(62.8)	
	**Yes**	18(39.1)	3(27.3)		5(27.8)	16(41)		16(37.2)	1(33.3)	1(33.3)	3(100)		3(50)	2(25)	16(37.2)	

TableS5: Association between clinicopathological characteristics and KRAS and NRAS mutations

	KRAS status									NRAS status								
	Exon 2 N=57		*p*	Exon 3 N=50		*p*	Exon 4 N=51		*P*	Exon 2 N=47		*p*	Exon 3 N=57		*p*	Exon 4 N=44		*P*
	M	WT		M	WT		M	WT		M	WT		M	WT		M	WT	
Gender			0.189			★			0.383			0.447			0.439			★
**Female**	7(43.8)	16(39)		0(0)	28(56)		3(75)	25(53.2)		0(0)	26(56.5)		0(0)	32(57.1)		0(0)	23(52.3)	
**Male**	9(56.3)	25(61)		0(0)	22(44)		1(25)	22(46.8)		1(100)	20(43.5)		1(100)	24(42.9)		0(0)	21(47.7)	
Age			0.285			★			0.161			0.574			0.596			★
**<50**	5(31.3)	18(43.9)		0(0)	21(42)		3(75)	17(36.2)		0(0)	20(43.5)		0(0)	23(41.1)		0(0)	17(38.6)	
**⩾50**	11(68.8)	23(56.1)		0(0)	29(58)		1(25)	30(63.8)		1(100)	26(56.5)		1(100)	33(58.9)		0(0)	27(61.4)	
Histologic type			0.636			★			0.779			0.936			0.947			★
**Adenocarcinoma**	15(93.8)	39(95.1)		0(0)	47(94)		4(100)	44(93.6)		1(100)	43(93.5)		1(100)	53(94.6)		0(0)	42(95.5)	
**Mucinous carcinoma/signet ring carcinoma**	1(6.3)	2(4.9)		0(0)	3(6)		0(0)	3(6.4)		0(0)	3(6.5)		0(0)	3(5.4)		0(0)	2(4.5)	
Degrees of differentiation			0.185			★			0.496			0.587			0.521			★
**Well**	11(68.8)	18(43.9)		0(0)	24(48)		1(25)	24(51.1)		0(0)	22(47.8)		0(0)	29(51.8)		0(0)	21(47.7)	
**Moderate**	5(31.3)	20(48.8)		0(0)	24(48)		3(75)	21(44.7)		1(100)	22(47.8)		1(100)	24(42.9)		0(0)	22(50)	
**poor/undifferenciated**	0(0)	3(7.3)		0(0)	2(4)		0(0)	2(4.3)		0(0)	2(4.3)		0(0)	3(5.4)		0(0)	1(2.3)	
Perineural invasion			0.660			★			0.347			0.106			0.123			★
**Yes**	1(14.6)	6(14.6)		0(0)	6(12)		1(25)	4(8.5)		1(100)	4(8.7)		1(100)	6(10.7)		0(0)	5(11.4)	
**No**	15(93.8)	35(85.4)		0(0)	44(88)		3(75)	43(91.5)		0(0)	42(91.3)		0(0)	50(89.3)		0(0)	39(88.6)	
Vascular invasion			1.000			★			0.221			0.064			1.000			★
**Yes**	1(6.3)	4(9.8)		0(0)	4(8)		1(25)	2(4.3)		1(100)	2(4.3)		0(0)	5(8.9)		0(0)	3(6.8)	
**No**	15(93.8)	1(6.3)		0(0)	46(92)		3(75)	45(95.7)		0(0)	44(95.7)		1(100)	51(91.1)		0(0)	41(93.2)	

ypN	**N0 N+**			0.548			★			0.629			0.277			0.667			★
		11(68.8)	27(65.9)		0(0)	34(68)		3(75)	32(68.1)		1(100)	34(73.9)		0(0)	37(66.1)		0(0)	31	
		5(31.3)	14(34.1)		0(0)	16(32)		1(25)	15(31.9)		0(0)	12(26.1)		1(100)	19(33.9)		0(0)	13	
ypT	**T0**			0.252			★			0.203			0.884	0		0.861	0(0)		★
	**T1**	1(14.6)	6(14.6)		0(0)	6(12)		0(0)	5(10.6)		0(0)	5(10.9)		0	7(12.5)		0(0)	4(9.1)	
**T2**		0(0)	3(7.3)		0(0)	2(4)		1(25)	1(2.1)		0(0)	2(4.3)		0	3(5.4)		0(0)	1(2.3)	
	**T3**	5(31.3)	16(39)		0(0)	17(34)		2(50)	18(38.3)		0(0)	17(37)		1(100)	21(37.5)		0(0)	17(38.6)	
	**T4**	9(56.3)	16(56.3)		0(0)	24(48)		1(25)	22(46.8)		1(100)	21(45.7)		0	24(42.9)		0(0)	21(47.7)	
		1(6.3)	0(0)		0(0)	1(2)		0(0)	1(2.1)		0(0)	1(2.2)			1(1.8)			1(2.3)	
Recurrence	**No**	1(2.4)	40(97.6)	0.187	0(0)	47(94)	★	4(100)	44(93.6)	0.779	1(100)	43(93.5)	0.936	1(100)	53(94.6)	0.947	0(0)	41(93.2)	★
	**Yes**	14(87.5)	1(2.4)		0(0)	3(6)		0(0)	3(6.4)		0(0)	3(6.5)		0(0)	3(5.4)		0(0)	3(6.8)	
Metastasis				0.478						0.016			0.404			0.351			★
**No**		5(31.3)	26(63.4)		0(0)	32(64)	★	0(0)	32(68.1)		1(100)	28(60.9)		0(0)	37(66.1)		0(0)	27(61.4)	
	**Yes**	15(36.6)	11(68.8)		0(0)	18(36)		4(0)	15(31.9)		0(0)	18(39.1)		1(100)	19(33.9)		0(0)	17(38/6)	
Metastasis or/and recurrence				0.410			★			0.019			0.426			0.368			★
**No**		11(68.8)	5(31.3)		19(38)	31(62)		0(0)	31(66)		0(0)	27(58.7)		0(0)	36(64.3)		0(0)	18(40.9)	
	**Yes**	5(31.3)	16(39)		0(0)	0(0)		4(100)	16(34)		1(100)	19(41.3)		1(100)	20(35.7)		0(0)	26(59.1)	

★,p was not calculated because the variable is constant
